# Subepithelial nevus and papilloma of the lacrimal caruncle: a case report and literature review

**DOI:** 10.3389/fopht.2026.1766863

**Published:** 2026-07-13

**Authors:** Siriphat Daocharoenporn, Weerawan Chokthaweesak, Chawawat Kangwanwongpaisan, Siroratt Narkcham, Bunyada Putthirangsiwong

**Affiliations:** 1 Department of Ophthalmology, Faculty of Medicine Ramathibodi Hospital, Mahidol University, Bangkok, Thailand; 2 Department of Pathology, Faculty of Medicine Ramathibodi Hospital, Mahidol University, Bangkok, Thailand

**Keywords:** caruncle, caruncle lesion, lacrimal caruncle, nevus, papilloma

## Abstract

**Background:**

Lacrimal caruncular tumors are rare and exhibit a diversity of possible histopathological types. The majority of caruncular tumors are benign, followed by premalignant and malignant lesions, which are associated with a poorer prognosis. Clinical diagnosis is challenging. Therefore, a tissue biopsy is considered and performed based on the presence of features suggesting malignant or undetermined tumors for a definite diagnosis and proper management. The coexistence of different types of benign caruncular tumors is extremely rare and has been rarely described.

**Case presentation:**

We herein describe the case of a 63-year-old Thai woman with a history of chronic progressive painless visual loss in her right eye who reported a non-progressive painless hyperpigmented mass without a history of associated contact bleeding on her left lacrimal caruncle. Ophthalmic examination revealed a large hyperpigmented choroidal mass with adjacent exudative retinal detachment in her right eye and a papilliform pinkish lesion over a well-defined, homogeneous, hyperpigmented mass on her left caruncle. Magnetic resonance imaging (MRI) scans of the orbit indicated a malignant choroidal melanoma in her right eye. The regional and distant metastatic work-ups were unremarkable. The patient was treated with a right globe enucleation concurrent with complete tumor removal of the left caruncular mass based on the presence of intratumorally dilated vessels with a feeding vessel to the overlying papilliform lesion, raising suspicion of malignancy, using the no-touch technique, followed by double freeze–thaw cryotherapy. Histopathological examination disclosed a choroidal melanoma in the right eye and a subepithelial caruncular nevus with an overlying conjunctival papilloma emerging from the conjunctival epithelium of the nevus in the left eye. The patient made a complete recovery with no evidence of recurrence at the 12-month follow-up.

**Conclusion:**

Coexistence of benign caruncular tumors is extremely rare. We report a rare case of a subepithelial nevus combined with a papilloma at the lacrimal caruncle. Complete tumor resection using the no-touch technique is the primary treatment. Adjunctive double freeze–thaw cryotherapy may be considered to further prevent tumor cell seeding and postoperative recurrences.

## Introduction

The lacrimal caruncle is a fleshy structure located between the plica semilunaris and the medial angle of the eyelid aperture. It evolves embryologically as a separation from the medial lower eyelid and is not formed from the conjunctiva (1, 2). It is composed of modified skin with pilosebaceous units, accessory lacrimal gland tissue, hair follicles, goblet cells, fibrofatty tissue, occasional smooth muscle fibers, and eccrine glands. Beneath the caruncle, there may be several large sebaceous glands without cilia. The epithelium of the caruncle is non-keratinized, stratified squamous epithelium similar to the conjunctival epithelium. These various types of tissue contribute to a wide variety of lesions arising from this specific area, leading to difficulty in definite clinical diagnosis (3–10). Despite its distinctive anatomy, the origin of the caruncle remains controversial due to its histological resemblance to both skin and conjunctival components. Therefore, the caruncular lesions are generally classified according to the tissue of origin found in histopathological examination.

Caruncular tumors are rare, with a relative frequency ranging from 0.3% to 1.1% of all excised caruncular specimens (4–9). The majority of caruncular tumors are benign, accounting for 84.6%–97.6% of cases, whereas premalignant and malignant lesions are found in 0%–7.7% and 2.4%–7.7%, respectively. The most frequently reported caruncular benign tumors are nevi (21%–60%), followed by papillomas (7%–43%) (4–12).

The coexistence of different types of caruncular tumors is extremely rare. In a review of the literature, there were only three cases previously reported: one case of a subepithelial nevus with a papilloma (13), a second case involving an intradermal nevus with a papilloma (14), and a third case of caruncular sebaceous gland hyperplasia with a papilloma (15). Herein, we describe a case of a caruncular subepithelial nevus with a coexisting papilloma.

## Case presentation

A 63-year-old Thai woman, a retired non-smoker, with a medical history of epilepsy and deep vein thrombosis in her left leg, presented with progressive, painless visual loss in her right eye over the past month prior to arrival at our hospital. She also reported a non-progressive, painless hyperpigmented mass without a history of associated contact bleeding on her left lacrimal caruncle. The mass had been first noticed approximately 20 years ago. Before this visit, she had not recognized an elevated whitish lesion overlying the pigmented mass. She denied any history of previous periorbital trauma, chemical injury, or surgery, and her family history was unremarkable. Notably, the patient reported a habit of eye rubbing, which, although not excessive, was more frequent than usual.

**Figure 1:**
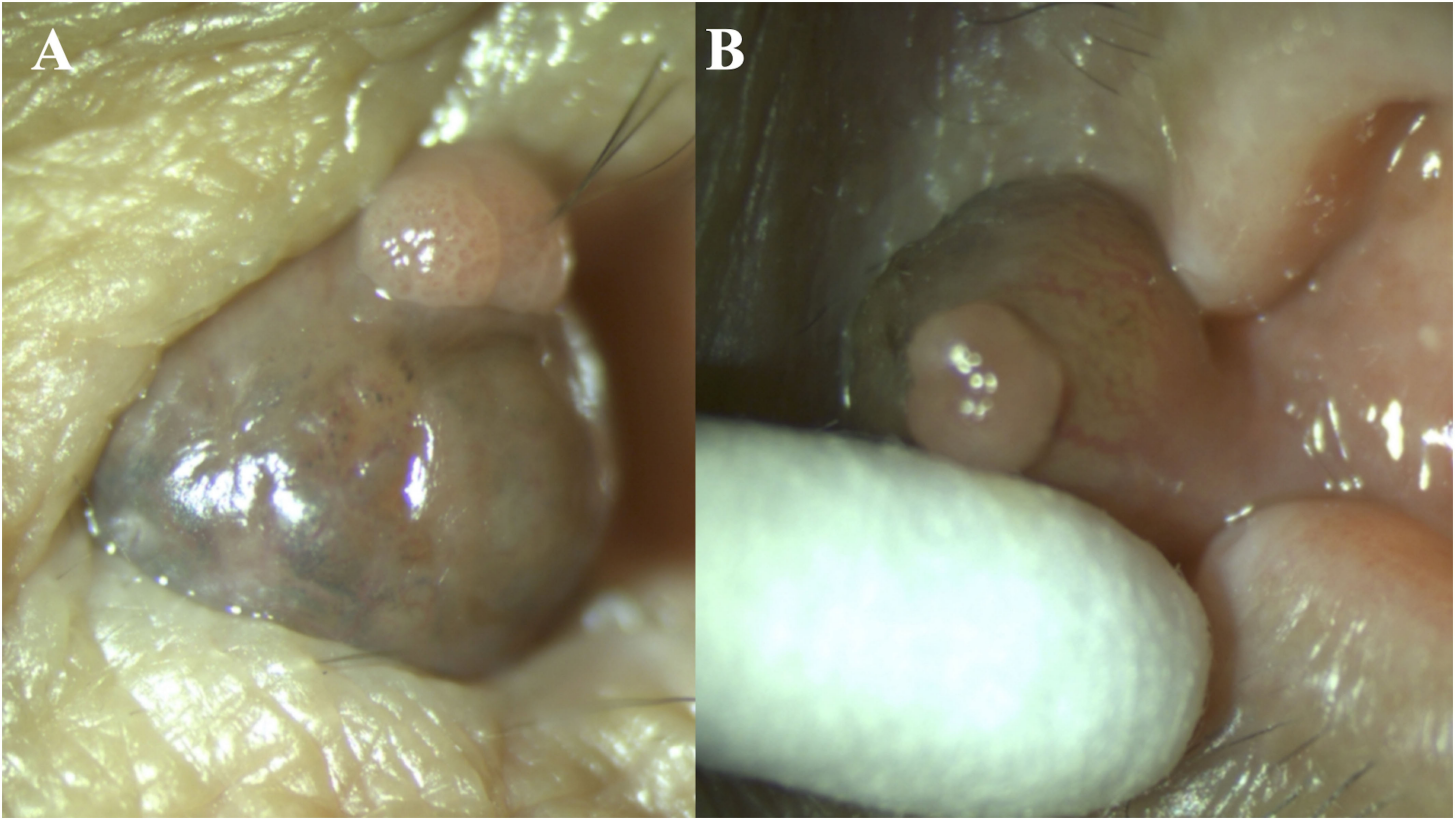
Clinical appearance of the tumor of the left caruncular mass at the first visit. **(A)** A papilliform pinkish lesion over a well-defined, smooth surface, hyperpigmented mass with intratumorally dilated vessels. **(B)** A feeding vessel to the overlying papilliform lesion on the left caruncle.

Ophthalmic examination revealed hand motion in the right eye and 20/20 in the left eye with a positive relative afferent pupillary defect (RAPD) in her right eye. Slit lamp examination revealed a papilliform pinkish lesion over a well-defined, smooth surface, a hyperpigmented mass with intratumorally dilated vessels, and a feeding vessel to the overlying papilliform lesion on the left caruncle (**Figure 1**). The anterior segments of both eyes were otherwise within normal limits, and no other melanocytic lesions were observed on the remaining conjunctiva or other mucosal surfaces. Fundus examination of the right eye revealed a large hyperpigmented choroidal mass with adjacent exudative retinal detachment; findings were unremarkable in the left eye. The extraocular movements of both eyes were full in all directions.

Magnetic resonance imaging (MRI) of the orbit demonstrated a hyperintense T1, hypointense T2 choroidal mass of the right globe, which was highly suggestive of malignant choroidal melanoma. The regional and distant metastatic work-ups were performed and found to be unremarkable.

The differential diagnosis for the left caruncular mass encompassed both benign entities (such as nevus, primary acquired melanosis, and papilloma) and malignant neoplasms (including malignant melanoma, squamous cell carcinoma, and sebaceous gland carcinoma). Given the atypical clinical presentation of dual tumor characteristics and the inability to definitively exclude malignancy based solely on physical examination, a complete excisional biopsy was mandated to establish a definitive histopathological diagnosis.

The patient underwent enucleation of the right globe concurrently with a complete excisional biopsy of the left caruncle mass, utilizing a no-touch technique under general anesthesia. To avoid tissue distortion or tumor cell seeding, the procedure was performed without injecting additional local anesthetic into the surgical site. Forceps were used to grasp the normal tissue adjacent to the base of the lesion, completely avoiding direct manipulation of the tumor itself until en-bloc excision was accomplished. The enucleated right globe and the excised left caruncular mass were submitted to the Department of Pathology as two separate specimens. Notably, the hyperpigmented mass and its overlying papilliform structure were not separated during the procedure; rather, they were excised and submitted together as a single en-bloc unit. Following the excision, double freeze–thaw cryotherapy was applied to the edges of the remaining conjunctiva. The surgical site of the left eye was then closed using a simple closure technique.

**Figure 2:**
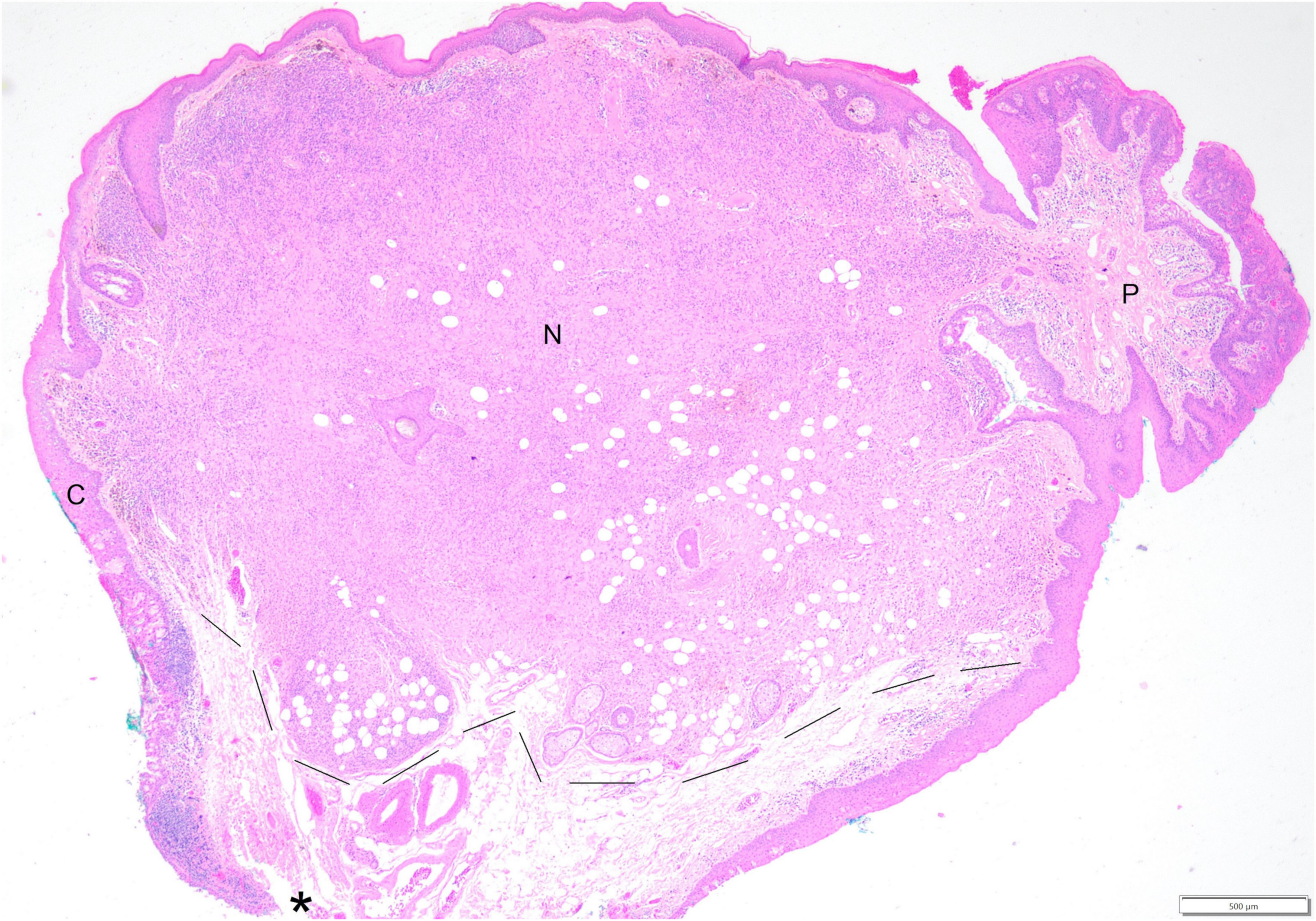
A mass at the caruncle of the left eye. A small conjunctival papilloma (P) arising on a subepithelial nevus (N), which is covered by conjunctival epithelium (C). The entire lesion (above the dashed line) is completely excised (the edge of the resection margin is marked with an asterisk) (hematoxylin and eosin, ×20; scale bar, 500 µm).

**Figure 3:**
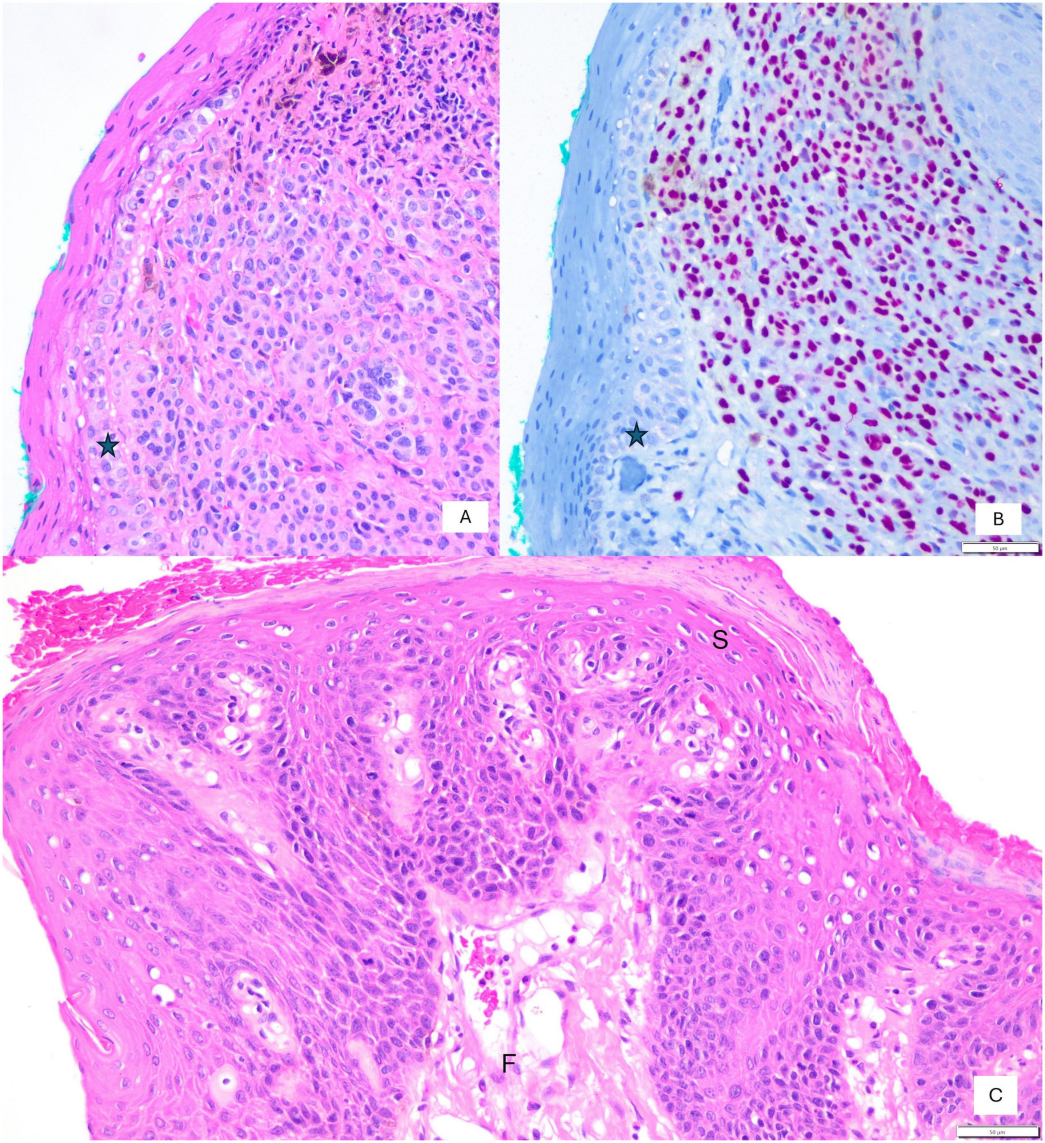
Caruncular nevus and squamous papilloma. **(A)** A suspected area (star) of melanocyte involvement in the epithelial layer, mimicking a compound nevus (hematoxylin and eosin, ×200; scale bar, 50 µm). **(B)** As the cells in this area (star) are negative for the melanocytic marker, the mass is consistent with a subepithelial nevus (SOX-10 immunostaining with red chromogen, ×200; scale bar, 50 µm). **(C)** A fibrovascular core (F) is covered by stratified squamous epithelium (S) with hyperkeratosis. Some koilocytes (indicative of HPV changes) are noted (hematoxylin and eosin, ×200; scale bar, 50 µm).

Gross examination of the caruncular mass revealed a 0.6 × 0.5 × 0.4 cm, hyperpigmented, polypoid nodule with a smooth mucosal surface, accompanied by a 0.2-cm-diameter tan nodule at the top of the hyperpigmented mass. Serial sectioning with perpendicular margins and formalin-fixed, paraffin-embedded (FFPE) tissue sections with hematoxylin and eosin (H&E) stain were performed. Diagnosis was established primarily based on characteristic H&E morphology, supplemented by SOX-10 immunohistochemistry to evaluate the melanocytic component. Microscopic findings of the hyperpigmented mass revealed a well-circumscribed, symmetrical lesion of melanocytic proliferation with maturation that was predominantly located within the substantia propria, consistent with a subepithelial nevus of the conjunctiva (**Figures 2**, **3A, B**). The covering epithelial layer comprised squamous epithelium containing goblet cells without dysplasia. A continuous, tiny nodule above the mass demonstrated radiating fibrovascular cores within the stroma, which was covered by stratified squamous epithelium with occasional koilocytes (indicative of human papillomavirus infection changes), as shown in **Figure 3C**. The excisional margins of the entire lesion were uninvolved by melanocytes.

Histopathological findings were consistent with a choroidal melanoma in the right eye and a combination of a subepithelial nevus with a conjunctival papilloma originating from the conjunctival epithelium of the nevus in the left eye.

At the 12-month postoperative follow-up, clinical examination revealed no evidence of tumor recurrence at either the left caruncle or the right anophthalmic socket. The patient expressed relief following the successful surgical excision and reported high satisfaction with the final cosmetic outcome. A comprehensive timeline detailing this episode of care is provided in **Table 1**.

**Table 1:** Timeline of the episode of care.

Timeframe	Clinical event and interventions	Findings and outcomes
~20 years prior to presentation	Patient first noticed a mass on the left eye.	A non-progressive, painless hyperpigmented mass on the left lacrimal caruncle.
1 month prior to presentation	Onset of new ocular symptoms in the right eye.	Progressive, painless visual loss in the right eye.
Initial presentation	Comprehensive ophthalmic examination, MRI of the orbit, and metastatic work-up.	Right eye (OD): hand motion vision, RAPD positive, choroidal mass with exudative retinal detachment (suggestive of melanoma). Left eye (OS): 20/20 vision, papilliform pinkish lesion overlying a hyperpigmented caruncular mass. Systemic: Metastatic work-up was unremarkable.
Surgical intervention	Conducted under general anesthesia.	OD: Enucleation. OS: Complete excisional biopsy (no-touch technique) with double freeze–thaw cryotherapy to conjunctival edges.
Postoperative pathology	Histopathological evaluation of the excised specimens.	OD: Confirmed choroidal melanoma. OS: Confirmed coexistence of a subepithelial nevus and a conjunctival papilloma.
12-month Follow-up	Clinical follow-up examination.	Patient remained stable with no evidence of tumor recurrence in either eye.

RAPD, relative afferent pupillary defect.

## Discussion

Preoperative clinical diagnosis of caruncular lesions remains challenging due to their histopathological diversity (11, 16), yielding an overall diagnostic accuracy rate of only 50% (4–6, 8–11). Although common benign tumors such as nevi and papillomas are identified with higher precision (4–12), the potential for malignant lesions with poor prognoses necessitates vigilant early detection. Consequently, definitive histopathological evaluation is crucial whenever features suggesting malignancy are present to ensure appropriate management and optimal clinical outcomes.

Conjunctival nevi are histologically categorized into three subtypes, including junctional, compound, and subepithelial nevi, based on the location of the nest of benign melanocytic cells (8, 17). Junctional nevi evolve from a benign proliferation of nevus cells located within the conjunctival epithelium along the epithelial and subepithelial junction. Compound nevi are proposed to grow from the junctional nevi, as the nevus cells protrude from the epithelium into the substantia propria, forming an intralesional epithelial cyst lined by goblet cells. Subepithelial nevi develop as the nevus cells in the conjunctival epithelium are lost, leaving a post-mitotic state of the benign melanocytic cells only in the substantia propria (17).

Conjunctival papilloma is an acquired benign epithelial tumor originating from the stratified squamous epithelium of the conjunctiva (18). Although the definite etiology of the conjunctival papilloma remains unclear, the main risk factor reported in the literature has been associated with human papillomavirus (HPV) infection (19). HPV deoxyribonucleic acid (DNA) has been detected in 44%–92% of conjunctival papillomas (19), with HPV-6 and HPV-11 being the most commonly identified subtypes (18, 20). There are insufficient data to support ultraviolet (UV) light exposure, smoking, or immunodeficiency as significant risk factors for conjunctival papilloma (19). The mode of ocular HPV transmission is mainly classified into three groups, including vertical transmission during delivery through an infected birth canal, ocular autoinoculation from contaminated hands, and reactivation of latent HPV infections (21–24). Karcioglu et al. demonstrated the presence of HPV types 16 and 18 DNA not only in epithelial tumors of the ocular mucosa but also in non-neoplastic lesions, and even in healthy conjunctiva (22). Conjunctival papillomas are mostly located nasally and inferiorly on the conjunctiva (18, 20). As HPV infection is a notable factor in conjunctival papilloma, the predilection of this distribution may be influenced by eye rubbing habits and the natural tear drainage pathway from the superotemporal fornix to the lacrimal lake. Tear flow may move HPV particles to the nasal and inferior conjunctiva, facilitating HPV autoinoculation (18, 20).

Combined benign caruncular tumors are rarely documented, with only three previous case reports. In 2001, D’Hermies et al. reported a 63-year-old male patient who underwent a curative lesional excision of the left lacrimal caruncle (13). Histopathological study disclosed a combination of two dissimilar lesions of an epithelial squamous papilloma and a subepithelial nevus. In 2017, Ishikawa et al. described a 39-year-old woman who presented with a raised whitish wart overlying a pigmented lesion on the left lacrimal caruncle (14). Histopathological findings revealed a subepithelial nevus and a conjunctival papilloma arising from the normal intact conjunctival epithelium of the nevus. In 2018, Miura-Karasawa et al. reported a case of coexistence of HPV-51-associated conjunctival papilloma overlying sebaceous gland hyperplasia (15). However, it is possible that the actual number of cases is underestimated because the tissue biopsies for histopathological diagnosis are not routinely performed in all patients, and several similar cases may remain unpublished.

We describe the rare coexistence of a subepithelial nevus and a conjunctival papilloma originating from the abnormal epithelium of the nevus on the lacrimal caruncle, found incidentally alongside a contralateral choroidal melanoma. In our case, we postulate that the development of a papilloma could have resulted from the prominent nevus’s environmental exposure, which facilitated contact with an HPV-contaminated finger during the patient’s reported habitual eye rubbing. Furthermore, the caruncle’s prominent anatomical position makes it a natural “trap” for tear-borne particles. The pre-existing elevated nevus created a focal point for mechanical irritation and viral inoculation from the tear lake. We hypothesize that these combined factors promoted dysplastic and neoplastic changes, eventually leading to the development of a secondary caruncular neoplasia overlying the preexisting lesion. However, it is important to emphasize that this proposed mechanism remains highly speculative. Without molecular confirmation such as HPV DNA testing, this hypothesis should not be overinterpreted.

While caruncular tumors are biologically distinct from choroidal melanoma and lack any pathological relationship, their simultaneous presentation in this case strongly underscores the necessity of a comprehensive bilateral ophthalmic examination—even when a patient initially presents with a seemingly localized, benign-appearing lesion.

The mainstay of treatment for conjunctival tumors is conservative treatment for small asymptomatic lesions involving a routine slit lamp examination with a photographic record every 3 to 6 months or 6 to 12 months, depending on the level of concern and the rate of tumor progression (9, 17, 19, 25). Any progressive changes in size, pigmentation, or vascularity of tumors are monitored. Conjunctival lesions with features suggesting malignancy should be excised for histopathological examination. When the surgical treatment is indicated, wide excision using a no-touch technique is recommended to prevent tumor cells from seeding, followed by double freeze–thaw cryotherapy to the surgical edges of the bulbar conjunctiva to prevent postoperative recurrences. Conjunctival defect coverage after tumor resection can be primary closure, conjunctival autograft, amniotic membrane graft, or even mucosal graft. The non-surgical treatment options for conjunctival papilloma are medications including interferon alpha-2B, mitomycin C, 5-fluorouracil (5-FU), and cimetidine, contributing not only to primary non-invasive management but also to postoperative adjuvant therapy. A pattern scanning laser photocoagulation, carbon dioxide laser therapy, and photodynamic therapy are also options for alternative treatment (19). Three cases in the literature underwent complete tumor resection without concurrent cryotherapy, all yielding excellent outcomes without recurrence. In our patient, we performed a complete excisional biopsy using the no-touch technique, followed by adjunctive double freeze–thaw cryotherapy to the conjunctival surgical edges to maximize the prevention of postoperative recurrences. While there is currently no evidence of recurrence at the 12-month follow-up, the absolute necessity of this adjunctive cryotherapy warrants further evaluation in larger studies.

The strengths of this study include the high-resolution clinical and histopathological documentation of this rare pathology, alongside a 12-month postoperative follow-up—the longest observation period reported in the literature for this combination of caruncular tumors. However, certain limitations should be considered. Foremost, the clinical observations and postulated mechanisms derived from a single case report limit broad generalizability. Additionally, while characteristic histopathological findings supported our hypothesis of an HPV etiology, confirmatory HPV DNA subtyping was not performed. Lastly, although external examinations were unremarkable for other papillomatous lesions, the absence of systemic or cervical HPV screening means that concurrent mucosal infections cannot be entirely ruled out.

In conclusion, the simultaneous presentation of a subepithelial nevus and a conjunctival papilloma at the lacrimal caruncle is an exceptionally rare clinical entity. Complete excisional biopsy utilizing a no-touch technique remains the definitive diagnostic and therapeutic approach, with adjunctive cryotherapy serving as an optional management to further minimize recurrence risk.

## Data Availability

The original contributions presented in the study are included in the article/supplementary material. Further inquiries can be directed to the corresponding author.
